# Confirmation of absence: the need for advanced imaging of the left atrial appendage

**DOI:** 10.1186/s44348-024-00020-7

**Published:** 2024-06-25

**Authors:** Fernando Mané, Inês Conde, Rodrigo Silva, Mónica Dias, Sofia Fernandes, Cátia Oliveira, Catarina Vieira, Vítor Hugo Pereira

**Affiliations:** Cardiology Department, Hospital de Braga, Braga, Portugal

**Keywords:** Left atrial appendage, Cardiac computed tomography, Tranesophageal echocardiogram

## Case presentation

**Fig. 1:**
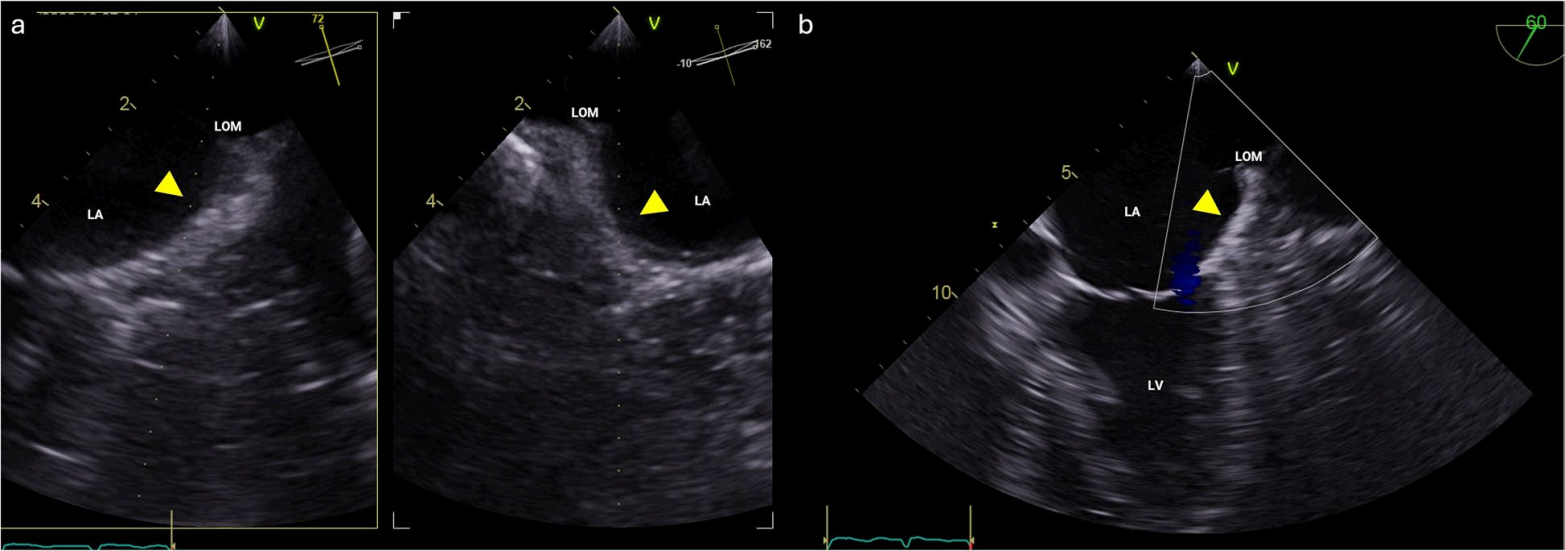
Initial three-dimensional (3D) transesophageal echocardiogram (TOE). **A** Magnified multiplane 3D TOE did not detect a patent left atrial appendage in its normal position (arrowheads). **B** Color Doppler unable to detect flow from the usual left atrial appendage location (arrowhead). LA, left atrium; LOM, ligament of Marshall; LV, left ventricle

**Fig. 2:**
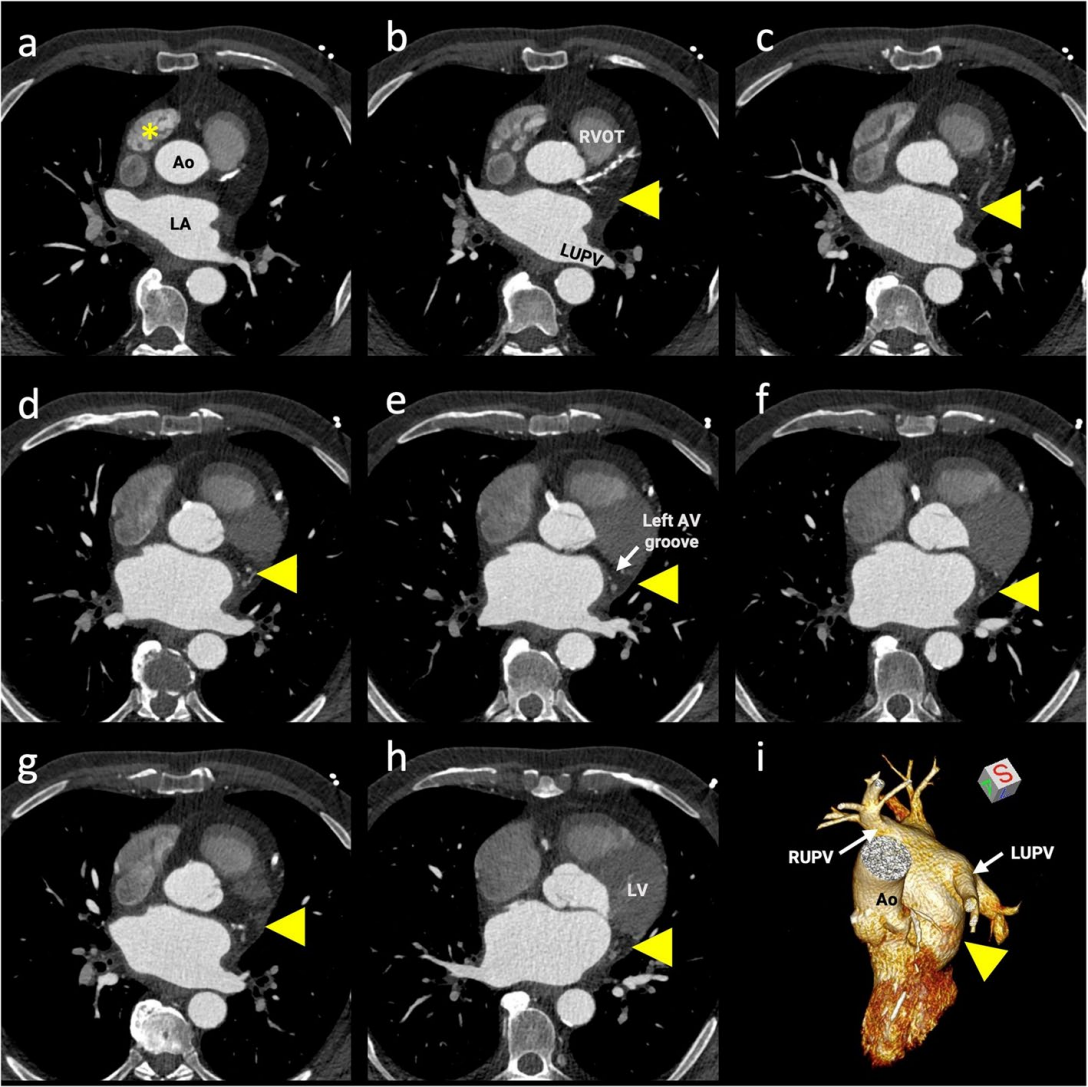
Cardiac contrast-enhanced computed tomography (CCT) images. **A**–**H** CCT axial-plane images cranial to caudal. Arrowheads show absence of the left atrial appendage in its normal position (arising from the anterolateral wall of the left atrium [LA] at the level of the left upper pulmonary vein [LUPV] and typically extending anteriorly, overlapping the left border of the pulmonary trunk and the circumflex artery). Asterisks represent the normally developed right atrial appendage. **I** Three-dimensional volume-rendered reconstruction of a left chamber comprehensively demonstrates the absence of the left atrial appendage (arrowhead). Ao, aorta; RVOT, right ventricular outflow tract; AV, atrioventricular; LV, left ventricle; RUPV, right upper pulmonary vein

A 65-year-old man with persistent atrial fibrillation was referred to our institute for catheter ablation treatment. Initial three-dimensional transesophageal echocardiogram using multiplane imaging failed to detect a patent left atrial appendage (LAA), and there was no Doppler flow detected from the usual LAA location (Fig. 1). Cardiac contrast-enhanced computed tomography (CCT) confirmed the congenital absence of LAA (Fig. 2). Pulmonary vein isolation was successfully achieved without complications, and anticoagulation was managed based on the CHA_2_DS_2_-VASc score.

## Discussion

While transesophageal echocardiogram remains the gold standard for identifying cardiac embolic sources, CCT can provide highly accurate imaging of the LAA if the appropriate scan algorithm, including a late pass scan, is employed [1]. Absence of the LAA is a rare finding, often incidentally discovered during preprocedural thrombus evaluation. In patients without a history of open-heart surgery, advanced imaging with CCT is crucial to distinguish between LAA absence and total thrombotic occlusion of the LAA. Decisions regarding anticoagulation in patients with LAA absence can be challenging due to limited evidence and the use of heterogeneous strategies [2].

## Data Availability

Not applicable.

## Abbreviations

CCT: Contrast-enhanced computed tomography
LAA: Left atrial appendage
